# Subclonal variant calling with multiple samples and prior knowledge

**DOI:** 10.1093/bioinformatics/btt750

**Published:** 2014-01-16

**Authors:** Moritz Gerstung, Elli Papaemmanuil, Peter J. Campbell

**Affiliations:** ^1^Cancer Genome Project, Wellcome Trust Sanger Institute, Hinxton, CB10 1SA, UK, ^2^Department of Haematology, Addenbrooke’s Hospital, Cambridge CB2 0QQ, UK and ^3^Department of Haematology, University of Cambridge, Cambridge CB22XY, UK

## Abstract

**Motivation:** Targeted resequencing of cancer genes in large cohorts of patients is important to understand the biological and clinical consequences of mutations. Cancers are often clonally heterogeneous, and the detection of subclonal mutations is important from a diagnostic point of view, but presents strong statistical challenges.

**Results:** Here we present a novel statistical approach for calling mutations from large cohorts of deeply resequenced cancer genes. These data allow for precisely estimating local error profiles and enable detecting mutations with high sensitivity and specificity. Our probabilistic method incorporates knowledge about the distribution of variants in terms of a prior probability. We show that our algorithm has a high accuracy of calling cancer mutations and demonstrate that the detected clonal and subclonal variants have important prognostic consequences.

**Availability:** Code is available as part of the Bioconductor package deepSNV.

**Contact:** mg14@sanger.ac.uk; pc8@sanger.ac.uk

## 1 INTRODUCTION

In recent years, genome sequencing has greatly enhanced our understanding of cancer biology (Stratton, 2011). Tumors are evolving entities and display complex clonal architectures with many mutations present in only a subset of cells (Nik-Zainal *et al.*, 2012; Yates and Campbell, 2012). Subclonal mutations provide insights into disease evolution and influence prognosis (Landau *et al.*, 2013; Papaemmanuil *et al.*, 2013). Subclonal variants can be detected using the deep coverage of next-generation sequencing technologies, but their distinction from sequencing errors, library preparation and alignment artifacts suffers from an unfavorable signal to noise level (Gerstung *et al.*, 2012; Schmitt *et al.*, 2012).

A series of powerful variant callers has been developed in recent years for calling variants from genome or exome sequencing data of tumor–normal pairs (Cibulskis *et al.*, 2013; Goya *et al.*, 2010; Larson *et al.*, 2011). For detecting subclonal variants, or mutations in samples with a low purity, which are both reported by small fractions of reads only, it is mandatory to accurately quantify the abundance of sequencing artifacts, which may otherwise lead to large numbers of false positives. With increasing numbers of genomic datasets being generated, it becomes apparent that sequencing artifacts tend to occur in a systematic way and on specific sites.

Targeted resequencing experiments, in which a selected set of candidate genes is resequenced across hundreds or thousands of samples, are increasingly prepared to evaluate findings from large-scale sequencing studies. Such datasets present an opportunity to precisely estimate the distribution of sequencing artifacts by aggregating information across samples, rather than across sites as is commonly done in tumor–normal variant calling. This will help avoid artifacts and likewise enable calling more variants on sites with lower error rates.

The growing catalogs of somatic mutations in cancer also make it possible to define genomic loci more likely to be mutated. Therefore, one may attempt to incorporate this prior knowledge to facilitate variant calling on mutational hotspots while remaining conservative on the remaining sites. Hence, a well-chosen prior will increase sensitivity at a given level of specificity.

Here we present a novel approach for detecting clonal and subclonal variants that exploits the power of a large sample set for precisely defining the local error rates and which uses prior information to call variants with high specificity and sensitivity.

## 2 APPROACH

Detecting mutations in deep sequencing data is essentially a model selection problem: one compares the probability of observing a given number of reads reporting a base change under a null model specifying the distribution of sequencing artifacts to the probability in an alternative model allowing for true variants. A mutation is called if the probability under the alternative exceeds that of the null model. A probabilistic framework offers the flexibility to account for prior information, which can be useful, as some genes are more likely to be mutated in particular cancers and there often exist mutational hotspots within a gene. The approach we present here for modeling the error distribution is based on the observation that sequencing artifacts are recurrent on specific loci. In a large cohort, this allows to define a background error distribution on each locus, above which true variants can be called.

## 3 METHODS

### 3.1 Statistical framework

To define a statistical test, we have to parameterize the distributions of variant allele counts. Let 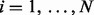 be the index of one of *N* samples. For each position *j* in the genome and nucleotide 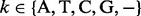, let *X_ijk_*, 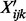 denote the count of that nucleotide in forward and backward read orientations in sample *i*. Let the coverage be denoted *n_ij_* and 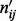, respectively. For the ease of reading, we omit the indexes *i*, *j* and *k*, unless a clear distinction is necessary. We model the nucleotide counts to be distributed by a beta-binomial distribution,
(1)
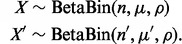



The parameters 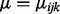 and 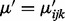 define the expected number of nucleotide counts per read,
(2)
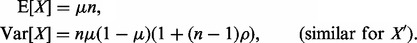



The dispersion factor 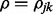 (no sample index) defines the amount of extra variance, as compared with pure sampling errors; for 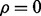, the model is the usual binomial.

Variant calling is commonly performed against a matched normal. Here we construct an aggregate control sample for sample *i* from the set of all other samples 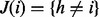, 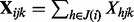 and 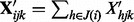 instead. The latter is justified if the particular variant occurs only rarely, or if the set of reference samples *J*(*i*) is chosen such that they are unlikely to contain the variant, e.g. by only selecting samples with a variant allele frequency (VAF) 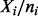 smaller than a predefined threshold, typically ∼10%. We assume that the control counts are also beta-binomially distributed with mean 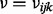, 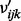 and coverage 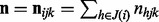 and 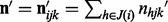:
(3)
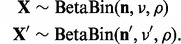



**Fig. 1.**
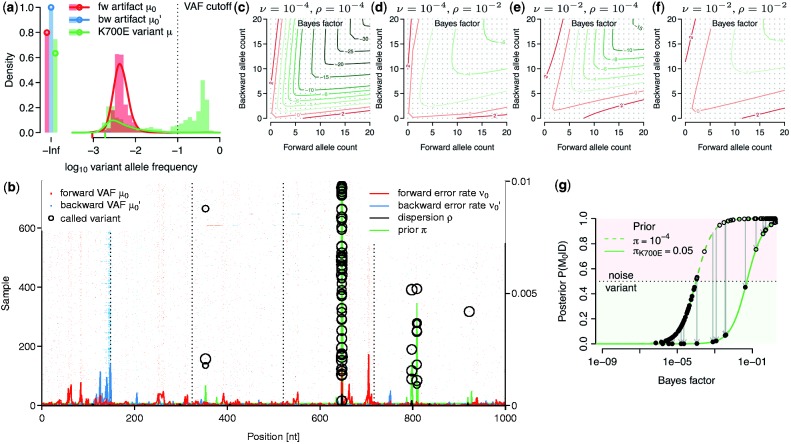
General illustration of our approach. (**a**) Distribution of observed and expected VAFs across samples. The histograms denote the VAF 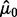 and 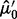 of a recurrent artifact occurring at low frequencies in ∼20% of the samples in forward, but not in the reverse orientation. The solid lines denote the expected distribution based on a beta-binomial model, Equation (1), with mean 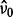 and 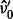 defined as the average across all samples with VAF 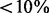. The third histogram denotes the *SF3B1* K700E variant present at clonal and subclonal frequencies, with the curve denoting the expected frequency distribution. (**b**) Heatmap of 1000 nt from five adjacent bait sets targeting the *SF3B1* gene in 683 samples. The intensity of each pixel represents VAF of cytosine, 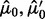, in a given sample (y, left axis) and position (x). If the relative frequency is identical, pixels tend to be black. Curves on the bottom indicate the error rates 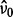 and 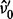 in forward and reverse directions (right y-axis). The black line is the estimated dispersion 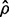. The prior π of finding a true variant is derived from the COSMIC database. Circles are drawn around variants with a posterior 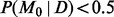; the area of each circle is proportional to the VAF. At position 650 resides the K700E hotspot mutation with many variant calls. (**c–f**) Bayes factors [Equation (7)] as a function of forward (x) and reverse (y) allele counts for different error rates 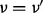 and dispersions 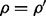. (**g**) A variant-specific prior π influences the Bayes factor needed to call a variant at a given cutoff on the posterior probability, Equation (9)

This definition is consistent with the assumption that the individual samples are beta-binomially distributed, as long as the dispersion parameter ρ is small. The above parameterization is similar to the deepSNV algorithm (Gerstung *et al.*, 2012), but uses aggregate control counts 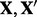 instead of a single control sample. We find that the model realistically reflects the observed distribution of nucleotide counts (Fig. 1a).

We formulate calling variants as a model selection problem. A true variant will be present on both strands, 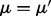, and at a higher frequency than both background error rates 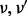 because it is the sum of the true allele frequency and the error rate. The null-model is that *X* and 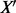 are distributed with the same rate as the control counts **X** and 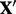 on either strand, which we assume to contain only errors but no variants. We then have the two models:
(4)
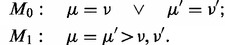



### 3.2 Inference

Denoting the data by 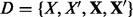, the Bayes factor 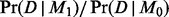 can be approximated using point estimates:
(5)
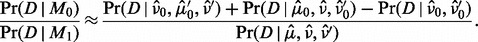



The three terms in the numerator arise from the OR condition of *M*_0_, Equation (4), and denote the probability that the error rates in forward, the reverse or both orientations are identical. Hence, the third term is usually small in cases where both allele frequencies 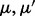 are different from the error rates. Note that this approximation is rather strong, but efficient to compute and works well in real applications. The point estimates are defined in the following way, using the method of moments:
(6)
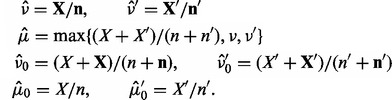



The symbols 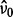 and 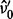 are the error rates across all samples; 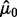 and 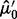 are the VAF in forward and reverse orientation for each sample (Fig. 1b).

The likelihood factorizes into 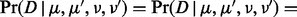

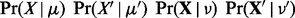
; this allows to write the Bayes factor as
(7)
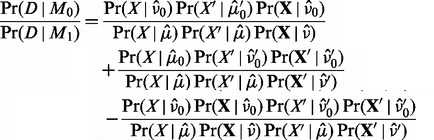



The value of the Bayes factor 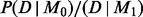 as a function of 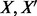 is illustrated in Figure 1c–f for different error rates. For a small error rate of 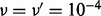, which is found on the majority of sites, only a few variant alleles lead to a Bayes factor small enough to call a variant.

### 3.3 Estimating *ρ*

There exists no closed-form solution to estimate *ρ*, but it can be estimated from the variances of the VAF 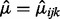 and total coverage 
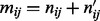
 across samples *i* by the method-of-moments estimator 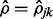:
(8)
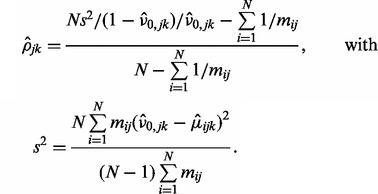



As this estimator is not guaranteed to yield values in (0,1), we bound it to 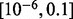. Empirically, we found that 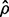 is usually small (Fig. 1b).

### 3.4 Prior data

The posterior probability that *M*_0_ is true can be computed by Bayes’ formula:
(9)
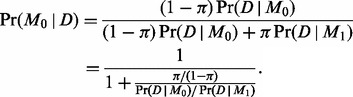



We use the probability of the null model *M*_0_ because of its similarity to a *P*-value in a hypothesis testing scheme and call variants below a certain threshold 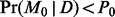. The parameter 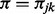 denotes the prior probabilities that a variant *k* exists at position *j*. The prior π essentially shifts the relation between the Bayes factor 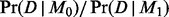 and the posterior probability 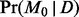. A higher prior probability results in a lower posterior probability of an artifact for a given signal as quantified by the Bayes factor (Fig. 1g).

Prior information about the likelihood of an allele being mutated can be extracted, for example, from the COSMIC database (Forbes *et al.*, 2011). We assume that the prior can be written as follows:
(10)
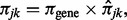

where the histogram
(11)
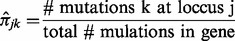

denotes the relative frequency of mutations *k* at site *j* in a given gene. The factor 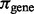 defines the probability of a gene being mutated. These probabilities vary greatly between genes and for the same gene also between different tumor types. As there are currently many systematic studies being performed, we expect that accurate estimates will be available soon for many cancers. For all sites not present in COSMIC, we use a constant value of 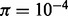. An example of the prior distribution obtained from COSMIC is shown in Figure 1b.

### 3.5 Implementation

We have implemented the algorithm in the statistical language *R* (R Core Team, 2012) and released code as part of the deepSNV Bioconductor package (≥1.8) (Gerstung *et al.*, 2012). We named the algorithm ‘shearwater’ after the seabirds that fly long distances over the ocean, watching the water closely and eventually dive into the water to pick up prey, often with prior help from other fish. More information can be found in the accompanying vignette:

> library(deepSNV)
> vignette(‘‘shearwater’’)

The runtime of 1 kb over 800 samples is ∼1 CPU min on a 2.2 GHz AMD processor. This performance is sufficient to process a complete targeted screen with 100 genes in a few hours on an 8-core machine, and the algorithm can be parallelized easily.

## 4 RESULTS

We benchmark our algorithm against data from two large gene screens in hematological cancers, a subset of 738 patients with myelodysplastic syndromes (MDS) we have published recently (Papaemmanuil *et al.*, 2013). In these screens, 111 cancer genes were sequenced using barcoded libraries prepared from whole genome amplified DNA. Samples were sequenced in batches of 96 per lane on a HiSeq2000 and reads were aligned with bwa (0.5.9 − r16 + rugo) (Li and Durbin, 2010) to the GRCh37 human reference genome. Technical replicates existed for 20 samples with acute myeloid leukemia (AML) assayed by the same gene panel. Moreover, we included 32 normal samples to quantify specificity. Here we focus on a subset of 43 genes with good coverage and in which we had previously found oncogenic mutations (Papaemmanuil *et al.*, 2013; Table 1). The availability of survival data in the MDS cohort allows for evaluating the quality of variant calls by their prognostic potential, which is an orthogonal measure to technical replication.

**Table 1.** Forty-three genes analyzed in 683 MDS samples with average coverage in parentheses

*ASXL1* (232), *ATRX* (393), *BCOR* (97), *BRAF* (415), *CBL* (392), *CDKN2A* (129), *CEBPA* (39), *CREBBP* (187), *CTNNA1* (309), *CUX1* (110), *DNMT3A* (94), *EP300* (370), *ETV6* (281), *EZH2* (470), *FLT3* (522), *GATA2* (38), *GNAS* (196), *IDH1* (341), *IDH2* (96), *IRF1* (61), *JAK2* (476), *KDM6A* (420), *KIT* (445), *KRAS* (274), *MLL2* (164), *MPL* (391), *NF1* (448), *NPM1* (345), *NRAS* (608), *PHF6* (236), *PTEN* (545), *PTPN11* (430), *RAD21* (330), *RUNX1* (247), *SF3B1* (282), *SH2B3*(113), *SRSF2* (65), *STAG2* (276), *TET2* (715), *TP53* (311), *U2AF1* (191), *WT1* (252), *ZRSR2* (197)

### 4.1 Simulations and control data

To assess the sensitivity and specificity of shearwater, we used a panel of 500 samples, including 32 normals and 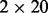 AML replicates. The remaining samples served for defining the background error distribution and for assessing how reproducible the calls are. To analyze the sensitivity for different combinations of coverage, we simulated mutations at different variant allele frequencies using the coverage and strand bias of one of the normal samples (median 128×, 5% 13×, 95% 372× coverage). For each position *j*, we drew a vector of variant allele frequencies for 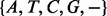 from a Dirichlet distribution, 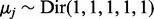. We then sampled reads 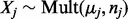, 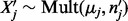, where *n_j_* and 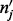 are the coverages on forward and reverse strand as observed in the normal sample. We ran shearwater on the cohort of 500 samples to compute the Bayes factors of each simulated variant.

#### 4.1.1 Sensitivity

**Fig. 2.**
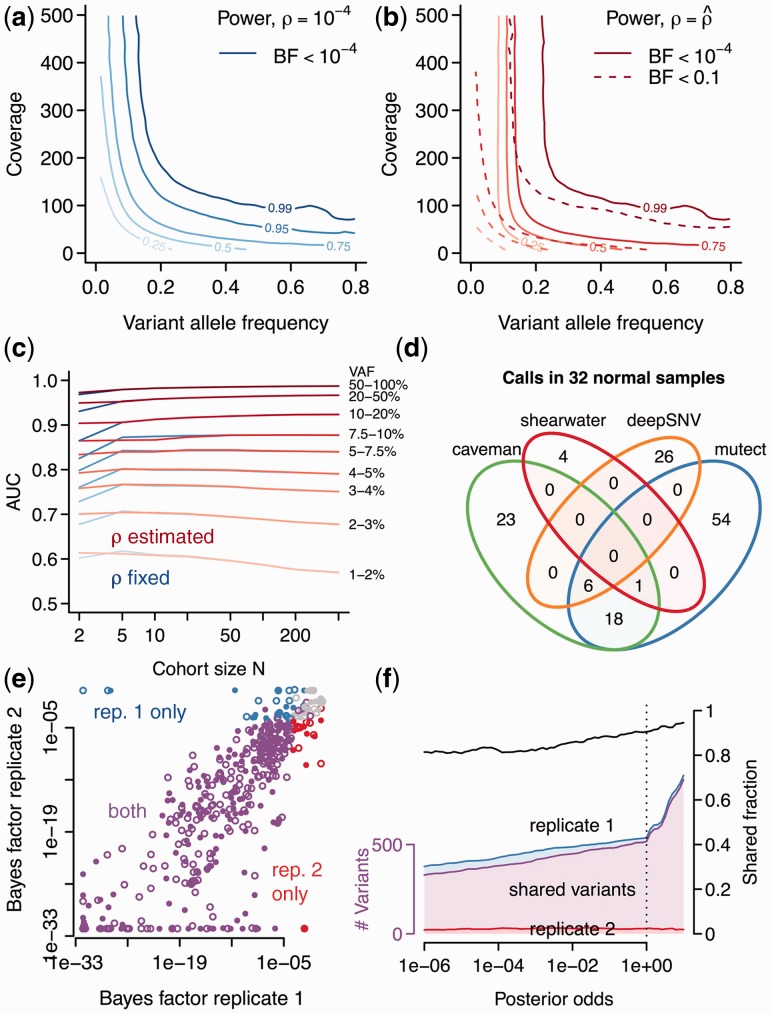
Variant calling in control data. (**a**) Power (true-positive rate) of detecting variants with different frequency and coverage for fixed dispersion *ρ*. (**b**) Power of detecting variants when *ρ* is estimated from the data using a VAF cutoff of 0.1. (**c**) AUC as a function of cohort size for different variant allele frequencies. The two lines for each VAF refer to the case 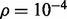 and to the case 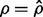, respectively. (**d**) Specificity of different algorithms on 32 normal control samples. (**e**) Scatterplot of Bayes factors for 20 replicates. Colors denote variants meeting a posterior threshold of 0.5 in only one of the two replicates. Open circles are known polymorphisms. (**f**) Concordance of variant calls as a function of the posterior cutoff. Filled segments show the number of variants called in either of the two replicates (top and bottom; left axis) and the overlapping fraction (middle) when a given posterior cutoff is applied. The black line (right axis) shows the relative proportion of overlapping to total calls

The fraction of variants with a Bayes factor <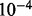 for fixed dispersion *ρ* is shown in Figure 2a. This cutoff corresponds to a posterior odds of 1, or a cutoff of 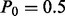, under a uniform prior with probability 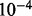. For a coverage of 250×, the true-positive rate of a 5% variant is 70%, and that of a 10% variant is ∼85%. Variants present in 20% can be called almost with certainty. When the dispersion is estimated from the data using all samples with VAF <10%, the Bayes factors become larger for variants <10% and only few reach the threshold of 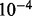, as the model starts fitting the distribution of true calls (Fig. 2b). In this case, a Bayes factor of 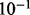 gives rise to a similar power as in the undispersed case and a stronger prior is required for a variant to be called.

#### 4.1.2 AUC and cohort size

We evaluated the area under the ROC curve (AUC) as a global measure of predictive accuracy for different VAF frequencies as a function of cohort size (Fig. 2c). Typical AUC values range from 60% for 1% variants to 98% for 50% VAF with only a mild influence of the cohort size. A small percentage of variants could not be called with the experimentally observed coverage.

#### 4.1.3 Specificity

As variants in cancer samples are typically rare and millions of loci are analyzed, specificity is a major concern. We compared shearwater’s specificity on 32 normal samples against three other algorithms: Caveman, an established variant caller, which has been used in many large-scale genome and exome sequencing projects (Jones *et al.*, unpublished data; Nik-Zainal *et al.*, 2012; Stephens *et al.*, 2012), MuTect (Cibulskis *et al.*, 2013) and deepSNV (Gerstung *et al.*, 2012). We ran Caveman as described against a single unmatched normal sample (Papaemmanuil *et al.*, 2013). Similarly, we ran MuTect (v.1.1.4) with default options —–cosmic b37_cosmic_v54_120711.vcf and —–dbsnp dbsnp_132_b37.leftAligned.vcf.gz against the same unmatched normal. The options of deepSNV (v.1.3.3) were combine.method=‘fisher’ and adjust.method=‘BH’. After calling variants, we filtered the output by removing variants in Ensembl variation (v70) and removed unknown polymorphisms with 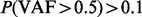.

In total, shearwater called five non-polymorphic variants (Fig. 2d). deepSNV, in contrast, called of 32, Caveman 48 and MuTect 79 variants. Hence, the specificity of shearwater appears satisfying.

#### 4.1.4 Reproducibility

To quantify the reproducibility of shearwater, we evaluated 20 AML samples that had been sequenced in replicates. Here the second replicate underwent whole-genome amplification, whereas the first replicate did not. The Bayes factors of replicates are highly correlated (Spearman’s 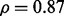) with only few samples missing the thresholds for variant calling (Fig. 2e). The overall overlap of variants called in both replicates ranges from 80 to >90%, depending on the posterior cutoff (Fig. 2f). This is consistent with an average power of 90–95%.

### 4.2 Variants in MDS

Here we reanalyze data from 683 MDS samples that were sequenced in the same run and passed quality control steps. We used the shearwater algorithm to analyze 258 830 nt from 43 oncogenic genes. For each call, we annotated polymorphisms present in Ensembl variation (v70) but not in COSMIC (v63) and termed mutations that were missense, nonsense or splice-site variants as non-silent.

#### 4.2.1 Effect of prior and cutoff

**Fig. 3.**
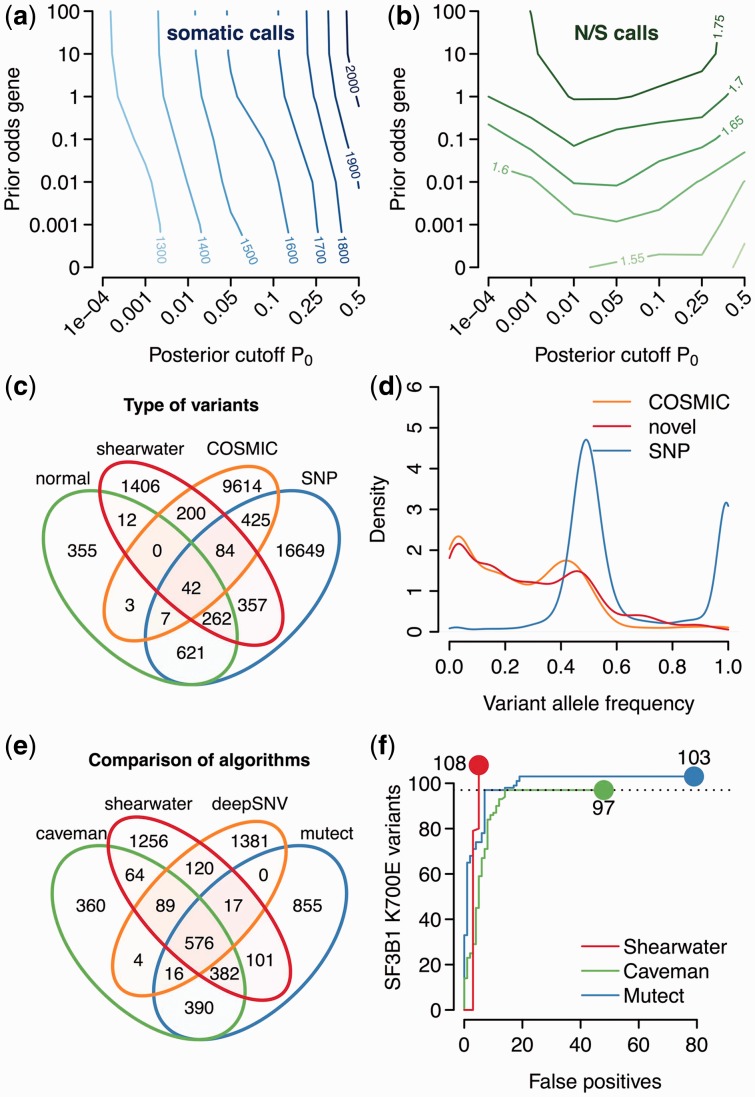
Variants in MDS. (**a**) Number of non-polymorphic variant calls versus cutoff *P*_0_ and prior weight 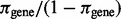. (**b**) Ratio of non-silent to silent variant calls. (**c**) Venn diagram of the distribution of shearwater variants across a normal panel, known SNPs and COSMIC variants. (**d**) Distribution of variant allele frequencies. (**e**) Venn diagram of calls from different algorithms. (**f**) Number of *SF3B1* K700E calls as a function of false positives for different variant callers

First, we assess the influence of the prior 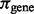 and the cutoff of the posterior probability *P*_0_, below which we call variants. As expected, the number of non-polymorphic variants calls grows when increasing either the cutoff of the posterior error probability *P*_0_, or the prior odds 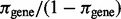 (Fig. 3a). As the cutoff *P*_0_ affects all sites, it has a somewhat larger influence on the number of calls than the prior, which affects only a small subset of sites.

A stronger prior weight, but not a larger posterior cutoff, leads to a higher ratio of non-silent to silent non-polymorphic calls (Fig. 3b), as the prior specifically enriches for non-coding variants. The absolute value of 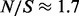 being smaller than the neutral value of ∼3 indicates that there may be some residual single-nucleotide polymorphisms present in the data (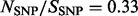).

In the following, we use a prior probability 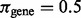 for a gene to be mutated in our cohort, which seems plausible, given that we resequenced cancer genes. We use a posterior cutoff of 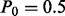 for our calls, which is the natural Bayes cutoff.

#### 4.2.2 Distribution of calls

With these parameters, shearwater made 20 975 calls across all samples, of which 2363 were unique variants (identical alleles present in multiple samples). Of these unique variants, 757 were found either in Ensembl variation or in an in-house panel of 500 normal exomes. Two hundred variants were present in COSMIC, but not in Ensembl variation, and 1406 were new (Fig. 3c).

The distribution of variant allele frequencies of known polymorphisms has two narrow peaks at 0.5 and 1, confirming the accuracy of allele frequency estimates (Fig. 3d). Non-polymorphic calls have a broad distribution with typical frequencies ranging from 0 to 0.5, with slightly more mass toward lower frequencies. This is consistent with our expectation that more variability exists at lower frequencies. The distributions of COSMIC and new variants are similar, which gives us confidence that these are real. This also indicates that the prior did not lead to overcalling, which would occur specifically at low frequencies.

#### 4.2.3 Comparison with other variant callers

We ran Caveman, MuTect and deepSNV against an unmatched normal as described above. After filtering variants from Ensembl variation, 576 variants were called by all four approaches (Fig. 3e). One thousand two hundred fifty-six variants were unique to shearwater, compared with 360 for Caveman, 1381 for deepSNV and 855 for MuTect. Four hundred five of 1256 unique variants were single base deletions, which could not be called by Caveman or MuTect. It therefore appears that shearwater achieves a good level of specificity, given that Caveman used a series of post-processing filters, whereas deepSNV and MuTect did not.

In the presence of noise, variant calling amounts to balance sensitivity and specificity. We evaluated this trade-off by comparing the ability for calling the *SF3B1* K700E hotspot mutation, which is characteristic of MDS and can thus be considered true somatic, versus the overall number of false-positive calls in the normal panel as discussed in the previous section. All three variant callers detect 97 K700E variants; above this level, however, MuTect and Caveman begin to call many artifacts (Fig. 3f). Shearwater calls 108 variants without decreasing specificity because of the higher prior weight (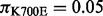) put on this variant. Yet no K700E variants were found in the normal samples, showing that shearwater does not blindly call this hotspot.

### 4.3 Prognostic performance

In the absence of a known ground truth and reliable methods for validating subclonal mutations that are guaranteed not to replicate systematic artifacts, it is generally difficult to assess the quality of one variant caller over another (Kim and Speed, 2013). An indirect measure of the quality of a predicted genotype can be the correlation with a known phenotype, such as survival. Suppose there exists a correlation *C* between genotype 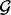 and a quantitative trait *Y*. In practice, we do not know the genotype with certainty, and only have estimates 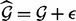, where *ε* is the deviation of the estimate from the truth. If *ε* is 0, the observed correlation between genotype and phenotype is 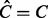; if *ε* is large and completely randomizes 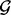 then the observed correlation becomes 0. Conversely, a higher correlation between genotype estimates and phenotype indicates a lower average bias of the genotype estimates. This reasoning requires the error *ε* and the phenotype *Y* to be uncorrelated and it appears unlikely to us that the ability to call mutations is confounded with the outcome of the patient in such a way that it leads over- and undercalling of mutations in specific sets of genes.

#### 4.3.1 Marginal effects of single genes

Survival in MDS depends on the absence and presence of mutations in multiple genes. For example, we and others have shown previously that oncogenic mutations in the *SF3B1* gene are associated with better prognosis (Damm *et al.*, 2012; Malcovati *et al.*, 2011; Papaemmanuil *et al.*, 2011), whereas alterations in *TP53*, *DNMT3A*, *STAG2* and other genes are indicative of a worse outcome (Papaemmanuil *et al.*, 2013). Patients with any novel mutations should hence follow these survival trends.

**Fig. 4.**
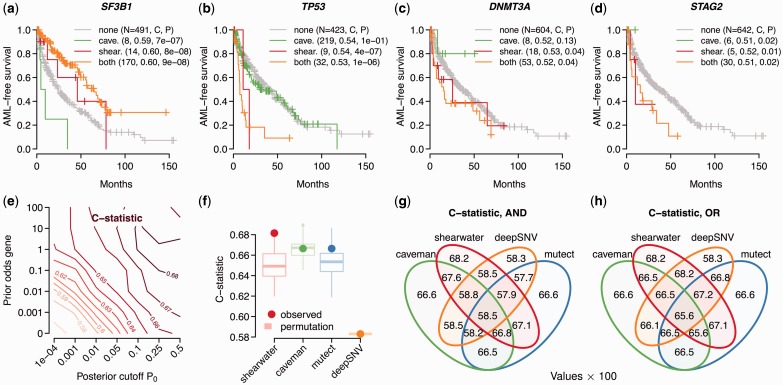
Prognostic effect of different variant callers. (**a–d**) The fraction of AML-free patients (either death or AML transformation) versus the time in months after sampling is shown. Patients are split into groups depending on whether the patient has a non-silent mutation in the given gene, found exclusively by Caveman, by shearwater only or by both. The gray line denotes patients with no mutations. *P*-values in the caption are from a log-rank test against the wild-type group, *C* is the corresponding *C*-statistic. While the Kaplan–Meyer curves and *N* refer to the fraction of patients exclusive to each method, *P* and *C* include the joint cases. (**e**) *C*-statistic for shearwater for different parameters. (**f**) *C*-statistic under permutation tests shuffling all calls in the set of variants exclusive to one variant caller. (**g**) *C*-statistic for different AND combinations of genotypes. (**h**) *C*-statistic for different OR combinations of genotypes

Survival data were available for 517 patients. We considered a gene to be mutated if it contained at least one non-silent mutation; the endpoint was AML-free survival. Figure 4a–d shows Kaplan–Meyer curves for patients carrying mutations identified by Caveman and/or shearwater. Patients with mutations only detected by shearwater generally display the expected behavior—that is on average better survival if *SF3B1* was mutated, poor survival if *TP53* or *STAG2* were mutated and a moderate change for *DNMT3A*.

#### 4.3.2 Overall prognostic accuracy

To assess the overall prognostic power combining all mutated genes, we trained Cox proportional hazards survival models with mutated genes as covariates. We used a 5-fold cross-validation scheme to estimate Harrel’s *C*-statistic (Harrell *et al.*, 1996), measuring the correspondence of the estimated risk and the ordering of deaths, similar to an AUC statistic, on the remaining fifth. The predictive potential *C* increases with *P*_0_ and the prior odds, with typical values between 0.67 and 0.68 (Fig. 4e). For a prior weight of 100 and 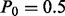, *C* starts dropping again as shearwater starts over-calling variants with a high prior probability. The maximal value of 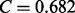 was observed for a cutoff of 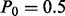 and a prior odds of 1, justifying our previous parameter choices.

The *C*-statistics of shearwater’s competitors were slightly lower, with Caveman having 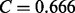 and permutations of the discrepant calls show that this difference is unlikely to be an artifact (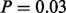; Fig. 4f). For Mutect we obtained 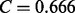 (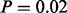) and deepSNV 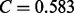 (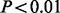). The higher prognostic accuracy of shearwater suggests that shearwater calls more survival-associated variants and less noise.

In a practical application, one will most likely rely on a combination of variant callers to avoid the biases of a single method. Combining the genotypes of different methods by either the intersection (AND) or the union (OR) of variant calls, however, did not further increase *C* (Fig. 4g, h). This indicates that the variants that shearwater may be missing do not have a large influence on survival.

## 5 DISCUSSION

In this article, we presented a statistical approach for detecting clonal and subclonal single nucleotide variants in targeted gene screens. The availability of large numbers of samples allows for precisely estimating the rate of artifacts, which is important for reliably detecting subclonal mutations that can have a disadvantageous signal to noise level. Our model incorporates prior information on mutational hotspots, which selectively increases the sensitivity for known mutations. Shearwater automatically determines the noise levels from the data, and we therefore expect it to deal well with sequencing data from other sequencing platforms and aligners.

Shearwater has both a high specificity and good power to detect variants. The genotypes obtained by shearwater have a higher prognostic value than those from established variant callers, and are likely to contain fewer artifacts. To an extent this behavior is expected because of our algorithm’s ability to exploit the power of a large cohort of samples and to incorporate prior knowledge about which mutations are more likely than others.

As our algorithm uses unmatched samples, it relies on the quality of polymorphism databases such as Single Nucleotide Polymorphism Database or Ensembl variation, which can generally be expected to become better in the future. The same holds true for the quality of databases of somatic mutations that will get richer over time and contain more precise information about the mutational patterns in each cancer type. Here we used the same probability for each gene to be mutated, but once unbiased estimates for the mutation frequencies in each cancer type exist from systematic gene screens, one will be able to further improve the accuracy of our algorithm. The idea of using a prior for recurrent mutations may also be incorporated easily into other variant callers.

Finally, our core algorithm may also be improved in many ways. For example, one could account for base qualities by a weighted counting scheme, instead of a simple phred quality threshold. One limitation of our approach is its reliance on a variant to be present on reads from both directions due to the specifics of the null model *M*_0_. This was introduced as it greatly increases the specificity of calls, but leads to a decrease in power in regions with low coverage and also at the flanks of the target regions, where often reads in only one direction are available. Our implementation allows the user to choose an essentially strand-agnostic null model 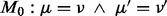, but this may be less specific. To analyze matched samples, one could derive the joint probability of a variant being present in only the tumor but not the normal, or simply remove the intersection of variants in tumor and matched normal.

In summary, we have presented a coherent statistical methodology and robust algorithm for calling subclonal variants in cancer samples with great specificity. As genomic sequencing is about to enter clinical diagnostics, we believe that our method will have broad applicability.

*Funding*: This work was supported by a Specialized Center of Research grant from the Leukemia Lymphoma Society (LLS), the Kay Kendall Leukaemia Fund and the Wellcome Trust (grant reference 077012/Z/05/Z). P.J.C. is personally funded through a Wellcome Trust Senior Clinical Research Fellowship (grant reference WT088340MA).

*Conflict of Interest*: none declared.
